# Supplementary material for: EnzML: multi-label prediction of enzyme classes using InterPro signatures
Source: BMC Bioinformatics. 2012 Apr 25;13:61. doi: 10.1186/1471-2105-13-61 (PMC3483700; doi:10.1186/1471-2105-13-61)
Supplement: Addtional file 5 — The Java code to format the data files, evaluate and predict. The file enzml_java_code.tar.gz contains the Java code used to format database data to ARFF and XML formats, to execute cross and train-test (jackknife) evaluations and to record evaluation results to database. More information is included in the readme.txt file and the Javadoc files. The code can be used with a MySQL database. To use a different database software, other JDBC drivers might be required. [file 1471-2105-13-61-S5.gz › java_code/enzml2011/doc/uk/ac/ed/inf/enzml/mulan/learn/MulanCrossExperimenter.html]

MulanCrossExperimenter


---


|  |  |  |
| --- | --- | --- |
| **Overview** \| **Package** \| **Class** \| **Use** \| **Tree** \| **Deprecated** \| **Index** \| **Help** | |  |
| **PREV CLASS**   **NEXT CLASS** | **FRAMES**    **NO FRAMES**     **All Classes** |  |
| SUMMARY: NESTED \| FIELD \| CONSTR \| METHOD | DETAIL: FIELD \| CONSTR \| METHOD |  |


---


## uk.ac.ed.inf.enzml.mulan.learn Class MulanCrossExperimenter

```
java.lang.Object
  uk.ac.ed.inf.utils.Initialised
      uk.ac.ed.inf.enzml.mulan.learn.MulanCrossExperimenter
```

**All Implemented Interfaces:**: uk.ac.ed.inf.utils.Initialisable

**Direct Known Subclasses:**: TrainTestExperimenter

---

``` public class MulanCrossExperimenter extends uk.ac.ed.inf.utils.Initialised ```

Using Mulan (multi-label machine learning), this class initialises and
executes either Train-Test evaluation (if two different arff records are
given) (calling `MulanTrainTestEvaluator`) or cross-evaluations (calling
`MulanCrossEvaluator`) if only one arff record is given. Initialisation
includes verifying input (arff and xml files) and checking output locations
(for saving results to file and database).

**Version:**
:   14 Jun 2010

**Author:**
:   Luna De Ferrari luna.deferrari-at-ed.ac.uk

---

**Field Summary**

**Fields inherited from class uk.ac.ed.inf.utils.Initialised**

`m_initialisationIsCorrect`


**Constructor Summary**

`MulanCrossExperimenter(java.lang.String resultsDbPropsFile,
java.lang.String arffRecordDbProps,
int trainArffRecordId,
MulanLearner learner,
java.lang.String resultsFolder)`
  
          public MulanCrossExperimenter(String resultsDbPropsFile, String
arffRecordDbProps, MulanArffRecord trainArffRecord, MulanLearner learner,
String resultsFolder) { m\_trainArffRecord = trainArffRecord;
this.initialise(resultsDbPropsFile, arffRecordDbProps, learner,
resultsFolder); }


| **Method Summary** | |
| --- | --- |
| `java.util.Vector<uk.ac.ed.inf.utils.database.TableRow>` | `crossEvaluateAndSaveResults(int folds)`             Cross validates and saves results to database |
| `uk.ac.ed.inf.utils.database.DbManager` | `getExperimentDbManager()`             public MulanCrossEvaluator getEvaluator() { return m\_evaluator; } |
| `java.lang.String` | `getExperimentPropsFile()` |
| `MulanLearner` | `getLearner()`             public String getExperimentPropsPath() { return m\_experimentPropsFile; } |
| `ResultsSaver` | `getResultsSaver()` |
| `MulanArffRecord` | `getTrainArffRecord()` |
| `static void` | `main(java.lang.String[] args)` |
| `static void` | `run(int[] arffFilesIds, java.lang.String resultsDbPropsPath)`             Run a cross-evaluation of several ARFF files (using one or several algorithms, as needed) |
| `ResultsSaver` | `setResultsSaver()` |

**Methods inherited from class uk.ac.ed.inf.utils.Initialised**

`initialisationIsCorrect, setInitialisationIsCorrect`

**Methods inherited from class java.lang.Object**

`equals, getClass, hashCode, notify, notifyAll, toString, wait, wait, wait`

**Constructor Detail**

### MulanCrossExperimenter

```
public MulanCrossExperimenter(java.lang.String resultsDbPropsFile,
                              java.lang.String arffRecordDbProps,
                              int trainArffRecordId,
                              MulanLearner learner,
                              java.lang.String resultsFolder)
```

:   public MulanCrossExperimenter(String resultsDbPropsFile, String
    arffRecordDbProps, MulanArffRecord trainArffRecord, MulanLearner learner,
    String resultsFolder) { m\_trainArffRecord = trainArffRecord;
    this.initialise(resultsDbPropsFile, arffRecordDbProps, learner,
    resultsFolder); }


**Method Detail**

### crossEvaluateAndSaveResults

```
public java.util.Vector<uk.ac.ed.inf.utils.database.TableRow> crossEvaluateAndSaveResults(int folds)
```

:   Cross validates and saves results to database

---


### getExperimentDbManager

```
public uk.ac.ed.inf.utils.database.DbManager getExperimentDbManager()
```

:   public MulanCrossEvaluator getEvaluator() { return m\_evaluator; }

---


### getExperimentPropsFile

```
public java.lang.String getExperimentPropsFile()
```

---


### getLearner

```
public MulanLearner getLearner()
```

:   public String getExperimentPropsPath() { return m\_experimentPropsFile; }

---


### getResultsSaver

```
public ResultsSaver getResultsSaver()
```

---


### getTrainArffRecord

```
public MulanArffRecord getTrainArffRecord()
```

---


### setResultsSaver

```
public ResultsSaver setResultsSaver()
```

---


### main

```
public static void main(java.lang.String[] args)
```

---


### run

```
public static void run(int[] arffFilesIds,
                       java.lang.String resultsDbPropsPath)
```

:   Run a cross-evaluation of several ARFF files (using one or several
    algorithms, as needed)

    :   **Parameters:**: `arffFilesIds` - a list of ARFF file identifiers in the "arff" database table


---


|  |  |  |
| --- | --- | --- |
| **Overview** \| **Package** \| **Class** \| **Use** \| **Tree** \| **Deprecated** \| **Index** \| **Help** | |  |
| **PREV CLASS**   **NEXT CLASS** | **FRAMES**    **NO FRAMES**     **All Classes** |  |
| SUMMARY: NESTED \| FIELD \| CONSTR \| METHOD | DETAIL: FIELD \| CONSTR \| METHOD |  |


---
